# Active monitoring of adverse reactions following COVID-19 and other vaccinations: a feasibility study as part of the CoVaKo project

**DOI:** 10.1186/s40814-022-01088-y

**Published:** 2022-07-02

**Authors:** Nikoletta Zeschick, Lisette Warkentin, Thomas Kühlein, Philipp Steininger, Klaus Überla, Susann Hueber, Maria Sebastião

**Affiliations:** 1 Institute of General Practice, University Hospital Erlangen, Friedrich-Alexander-Universität Erlangen-Nürnberg (FAU), 91054 Erlangen, Germany; 2 Institute of Clinical and Molecular Virology, University Hospital Erlangen, Friedrich-Alexander-Universität Erlangen-Nürnberg (FAU), Erlangen, Germany

**Keywords:** COVID-19, Vaccination, Adverse reactions, Online survey, Healthcare staff, Vaccination centres, General practitioners

## Abstract

**Background:**

The *Corona-Vakzin-Konsortium* project (CoVaKo) analyses the efficacy and safety of COVID-19 vaccines in a real-world setting, as well as breakthrough infections in Bavaria, Germany. A subproject of CoVaKo aims to identify adverse reactions of the COVID-19 vaccine and compare these to adverse reactions of other vaccines in an online survey. In a preceding feasibility study, the study materials were tested for comprehensibility, visual design, and motivation to participate, as well as for their ability to be implemented and carried out in primary care practices and vaccination centres.

**Methods:**

We used a mixed-methods research design. First, three focus groups consisting of general population participants were organised to evaluate the study materials and survey. Second, a test roll-out was conducted in vaccination centres and primary care practices that involved implementing and quantitatively evaluating the online survey. Third, interviews were conducted with participating general practitioners and heads of vaccination centres four weeks after the test roll-out.

**Results:**

Parts of the information and registration form proved incomprehensible, specifically regarding the recruitment material and/or online survey. For example, headings were misleading given that, relative to other vaccinations, the COVID-19 vaccination was overemphasised in the title. Participants requested additional information regarding the procedure and completion time. Within 31 days, 2199 participants, who received either a COVID-19 vaccination (99%) or at least one of the control vaccinations (1%), registered for the study. Participants (strongly) agreed that the registration process was easy to understand, that the completion time was reasonable, and that the technical setup was straightforward. Physicians and heads of the vaccination centres perceived the study as easy to integrate into their workflow. The majority expressed willingness to participate in the main study.

**Conclusions:**

Our study indicated that identifying and documenting adverse reactions following vaccinations using an online survey is feasible. Testing materials and surveys provided valuable insight, enabling subsequent improvements. Participation from health professionals proved essential in ensuring the practicality of procedures. Lastly, adapting the study’s organisation to external fluctuating structures and requirements confirmed necessary for a successful implementation, especially due to dynamic changes in the nation’s COVID-19 vaccination strategies.

**Trial registration:**

The trial was retrospectively registered at the “Deutsches Register Klinischer Studien” (DRKS-ID: DRKS00025881) on Oct 14, 2021.

**Supplementary Information:**

The online version contains supplementary material available at 10.1186/s40814-022-01088-y.

## Key messages regarding feasibility

1) What uncertainties existed regarding feasibility?

- It was uncertain whether a sufficient number of vaccinated individuals could be recruited for an online survey, especially in case of control vaccinations, older individuals, and/or individuals without technical experience. It was also uncertain whether staff from vaccination centres and physician practices would be willing to distribute recruitment materials without monetary compensation.

2) What are the key feasibility findings?

- Due to the nation’s ever-changing COVID-19 vaccination recommendations, flexibility in the study procedure proved to be important. The public’s focus was predominantly on COVID-19 during this time, causing difficulties in recruiting individuals who had recently received a control vaccination. Successfully implementing and integrating this study into healthcare institutions’ existing processes and procedures can succeed if done with as little effort from them as possible.

3) What are the implications of the feasibility findings for the design of the main study?

- In terms of the online survey, built-in self-checks (vaccination, gender, age) would be useful to increase the quality of the data. To reach individuals after they have received a control vaccination, more primary care practices and in-house company physicians should be recruited. In addition, spreading announcements through newspapers and the radio could be useful. In regard to the recruitment of healthcare providers, the study procedure must be easy to implement and be adaptable in case of potential changes in the federal vaccination strategy.

## Introduction

The COVID-19 pandemic has affected the lives of nearly everyone. Vaccines are a promising remedy against it. Up to now, COVID-19 vaccines developed by BioNTech/Pfizer, Moderna, AstraZeneca, and Johnson & Johnson have been authorised by the European Medicines Agency (EMA). Pivotal studies on these vaccines have shown that they all possess high levels of efficacy and safety [1–3]. Given that the efficacy and safety of a vaccine significantly impact its acceptance within a population [4, 5], effective monitoring systems are important to ensure a secure and successful vaccination campaign. A reliable assessment of adverse reactions, especially severe events requiring medical attention, is required. Because the COVID-19 vaccine was developed unprecedentedly fast, an active examination of its side effects is warranted; this should be accompanied by a comparison with other commonly used vaccines to reduce the risk of bias. In Germany, as of October 19, 2021, 54.7 million people have been fully vaccinated against COVID-19 [6].

The *Corona-Vakzin-Konsortium* project (CoVaKo) analyses the efficacy and safety of COVID-19 vaccines and breakthrough infections in Bavaria, Germany [7, 8]. One part of the project, which is called the CoVaKo safety study, aims to identify adverse reactions in a real-world setting using an online survey. The observation group will include participants who have recently received a COVID-19 vaccination. The control group will include participants who have recently received other common types of vaccinations, e.g. vaccinations against influenza, shingles, or pneumococcus. Posters, flyers, and leaflets will be used to recruit participants at vaccination centres and primary care practices. Those who are 18 years or older and have been recently vaccinated will be asked to register for the study via a secure website using REDCap (Research Electronic Data Capture), hosted by the Universitätsklinikum Erlangen [9, 10]. When registering, participants will provide information on sociodemographic characteristics, their vaccination, and any comorbidities using a modified German version of the Self-Administered Comorbidity Questionnaire (SCQ-D) [11–13]. Within 18 weeks after their vaccination, participants will be asked to complete an online survey (up to five times) about perceived adverse reactions and side effects, particularly reactions that lead to (in- or outpatient) medical consultation, medication intake, and/or sick leave.

With a preceding feasibility study, we aimed to determine whether an online survey to identify adverse reactions following a vaccination is practicable. The objective involved (1) testing the recruiting material for comprehensibility, visual design, and motivation to participate; (2) evaluating the online survey for comprehensibility, visual design, relevance of questions, and technical implementation; and (3) assessing its practical implementation and realisation in vaccination centres and primary care practices.

## Methods

### Study design and procedure

A mixed-methods research design was used to combine elements of qualitative and quantitative research approaches in order to analyse the study’s strengths, weaknesses, opportunities, and threats (SWOT). Initially, the recruitment materials and the online survey were evaluated qualitatively. During the test roll-out, the online survey was evaluated quantitatively, while the feasibility was evaluated both quantitatively and qualitatively.

The reporting of the study is based on the STROBE (Strengthening the Reporting of Observational Studies in Epidemiology) recommendations [14], the Consolidated Standards of Reporting Trials (CONSORT) statement [15], and on the COREQ-Checklist (COnsolidated criteria for REporting Qualitative research) [16].

The following table explains the different steps that make up the project’s procedure (Table 1).

**Table 1:** Overview of the study’s mixed-method procedure

To evaluate	Methods and objectives	
	Qualitative approach	Quantitative approach
Recruiting materials	*Method*: Focus groups with (vaccinated) persons *Objective*: comprehensibility, visual design, motivation to participate	NA
Online survey	*Method*: Focus groups with (vaccinated) persons *Objective*: comprehensibility, visual design, relevance of questions, technical accuracy	*Method*: Online survey with vaccinated persons *Objective*: comprehensibility, visual design, relevance of questions
Feasibility through test roll-out	*Method*: Interviews with GPs and heads of vaccination centres *Objective*: practicability of implementation/realization	*Method*: Online survey containing additional textbox with vaccinated persons *Objective*: recruitment and participation rates

*NA* not available

### Qualitative evaluation of recruiting materials and online survey

First, the recruitment materials were created. The aim here was to develop recruitment materials that directly address those who are vaccinated. In this regard, we designed different images, using the corporate colours of the Universitätsklinikum Erlangen to show its affiliation. The materials should be easy to understand and use everyday language. Hence, we included various formulations (e.g. vaccine side effects vs. vaccine reaction vs. vaccine damage).

Before the test roll-out, participants qualitatively evaluated the recruitment materials and the online survey. In March 2021, NZ and MS conducted three online focus groups (each 120 min); these are two female researchers at the Institute of General Practice with profound knowledge in qualitative research. The only inclusion criterion defined was a minimum age of 18 years. Inclusion was independent of vaccination status, as this was not relevant for the evaluation. Exclusion criteria were lack of written consent and/or mental or cognitive impairments. Participants were recruited in the MVZ Eckental, a primary care practice associated with our institute (Institute of General Practice, Universitätsklinikum Erlangen). Snowball sampling was used to reach further participants (convenience sample). NZ and MS did not know the participants prior to collecting their data. The participants received all versions of flyers, leaflets, and posters, as well as the study’s information before the focus groups took place. In the focus groups, the researchers discussed all materials and asked questions regarding comprehensibility and readability, attractiveness of the visual design from the participants‘ perspective, and whether materials stimulated participants’ interest and motivation. The participants then completed the online survey using different case vignettes (Supplementary material). They evaluated the survey for technical errors, visual design, and readability on different devices (mobile phones, tablets, and laptops) and different popular Internet browsers. With the researchers, they also discussed whether the study’s design and procedure, as well as the inclusion criteria specified on the materials, were described in an easy-to-understand manner. The focus groups were audio recorded; NZ and MS also took field notes. After each focus group, the project team summarised the results. Based on their findings, adjustments were made to the materials and the online survey, which was then discussed by the third focus group’s participants.

### Quantitative evaluation of the online survey

The study was tested across primary care practices and vaccination centres, and study participants evaluated the online survey quantitatively. The inclusion criteria were written consent and a recent vaccination. The online survey on adverse reactions was followed by a number of evaluation questions. The online survey included questions about the registration process, the structure of the survey, comprehensibility, completeness, and importance of the questions. It also contained questions about the technical implementation and method of recruitment. Items were answered using a 5-point Likert scale from 1 = *strongly disagree* to 5 = *strongly agree*. The evaluation form included a text box for further comments. The test roll-out and data collection for the evaluation form started on April 17, 2021, and ended on July 14, 2021.

Quantitative data were also collected on the participation rates of vaccinated individuals. The targeted minimum recruitment rate for the feasibility study was 50 participants/group. If we can reach this target in 4 weeks, we estimate that 3000 participants/group can potentially be recruited within 9 months of the large-scale main study.

### Qualitative evaluation of the practicability of the implementation

Feasibility was qualitatively evaluated via interviews with general practitioners (GPs) and heads of COVID-19 vaccination centres (May 2021). We recruited vaccination centres in Central Franconia (a region in Bavaria), directly via email and primary care practices through the BayFoNet, the Bavarian Practice Based Research Network (https://bayfonet.de/en/). The centres and practices did not receive any form of compensation. The centres’ and practices’ staff handed out our leaflets to patients following those patients’ vaccination. No further explanation from the staff was necessary. Posters and flyers could be used to draw more attention. The GPs and heads of the vaccination centres qualitatively evaluated the study’s process 4 weeks after the test roll-out via telephone interviews (30 min) conducted by NZ and LW (female doctor in training/researcher at the Institute of General Practice). The interviewers (NZ, LW) took field notes during the interviews. Interviewees answered questions concerning the distribution of the material (e.g. who distributed and when; time required) and patients’ reactions to the said material (e.g. curiosity, aversion, lack of interest, etc.) to adjust material if necessary. The structure of the study, contact frequency to the study centre, motivation to participate in the main study, and suggestions for recruiting other practices/vaccination centres were also discussed in the interviews.

### Data analysis

#### Qualitative evaluation

Information gathered from the focus groups were roughly summarised in writing. NZ and MS then used the method of mapping to structure and visualise their statements. Categories from the focus groups were comprehensibility and readability, visual design, technical errors, and motivation to participate. Changes to the recruitment material and surveys were then derived.

Categories from the field notes of the interviews were distribution of the material, patients’ reactions, organisation, motivation to participate, and recruitment of other practices/vaccination centres. The project team discussed the results, not the participants.

#### Quantitative evaluation

The email addresses were checked for duplicates. If participants entered the same email address in two different registrations, the project team reviewed whether one person registered twice for the same vaccination or whether the person and/or vaccination differed. In the first case, the datasets were synthesised. In the case of different vaccinations or different persons with the same email address, both datasets were taken into consideration individually. Various plausibility tests were used to check whether the participants correctly understood the questions and the given answer format. The project team checked the data for implausible answers, e.g. only participants with an interval of 14 to 91 days between their first and second COVID-19 vaccination dose were included due to the vaccination strategy recommendations. To monitor for potential errors in sending out the invitation links for the subsequent surveys, it was checked whether the time span between an individual’s vaccination and the time they answered the survey was correct and whether the interval between two surveys was at least 7 days. Sociodemographic characteristics, information about the vaccines, and comorbidities are reported as a proportion of all patients with a valid registration form. A dropout rate over the observation period was estimated by the amount of fully completed surveys in relation to the email invitations sent to the participants remaining after the data selection and preparation process. Comorbidity in the form of a modified SCQ-D was calculated [12]. We performed data preparation and analyses, as well as created figures using R Statistical Software (version 4.1.0, R Foundation for Statistical Computing, Vienna, Austria).

Each participant’s first evaluation form was considered for the analysis. NZ and MS categorised the comments in the text box in MAXQDA Plus 2020. The categories were content, organisation, and technical implementation. The project team discussed the results, not the participants.

## Results

### Qualitative evaluation of recruiting materials and online survey

#### Study population

Fourteen participants took part in three focus groups (Table 2); two participants dropped out due to personal reasons that were not related to the study. Roughly, an equal number of women and men participated. Almost all of the participants were employed and had a university degree. One participant did not speak German as her native language.

**Table 2:** Sociodemographic characteristics of focus group participants

Baseline characteristics	***n***	%
*N*	**14**	100
Gender		
Female	08	057
Male	06	043
Diverse	00	000
Year of birth		
1995–2003	00	000
1996–1980	10	071
1981–1960	00	000
1961 and older	04	029
Employment		
Employed	10	072
In education	02	014
Unemployed	02	014
Retired	00	000
Other	00	000
Not specified	00	000
Education		
No degree	00	000
Lower certificate	00	000
Intermediate certificate	01	007
Complete apprenticeship	01	007
High school diploma	02	014
University degree	10	072
Not specified	00	000

Focus group participants were divided into three groups

#### Evaluation of recruiting materials

**Fig. 1:**
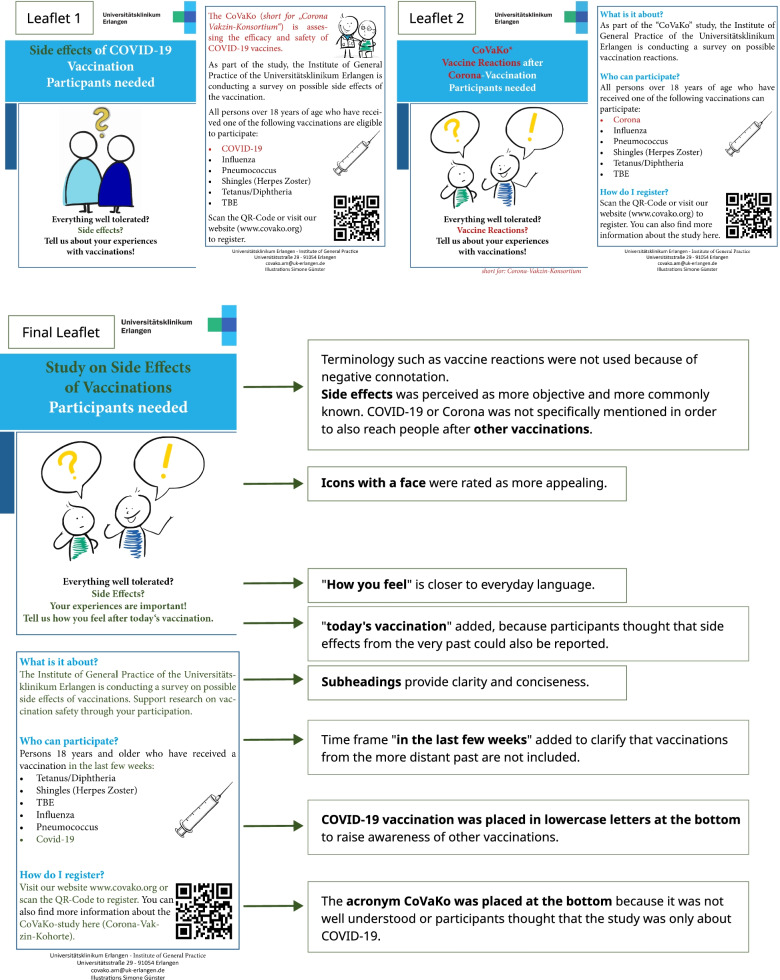
Leaflet before and after conducting focus groups. These are two out of five versions of the leaflet that were evaluated in the focus groups, as well as the final adjusted version that took feedback from the focus groups into account

The participants rated the leaflet that is the size of a vaccination certificate most positively, stating it is more practical compared to posters and flyers. Figure 1 shows two (out of five) different leaflets that were presented to the participants, as well as the final adjusted leaflet for the primary care practices.

In general, headlines were misleading because they only referred to the COVID-19 vaccination, not the control vaccinations. Participants wanted headings to be more reader-friendly, e.g. larger font. They were confused by the study acronym, CoVaKo, and preferred it not to be mentioned in the title. Some participants also preferred the masculine form of language compared to gender-neutral language that uses an asterisk (e.g. *Teilnehmer* instead of Teilnehmer*innen; *eng*. participants). They also favoured the wording *vaccine side effects* instead of *vaccine reaction* or *vaccine damage* since it is most commonly known and/or perceived as more neutral.

Information on the study should be listed as bullet points, one below the other, as opposed to alternating between left and right columns. Some participants preferred one image over two images on a page. Participants appreciated the information on data protection but found it too long. Some participants expressed wanting to know what will happen to the results and how data will be analysed. Most participants evaluated the options to register and access more information on the study via QR code or URL as practical. However, older participants stated they would not know how to use the QR code.

Final versions of the posters, leaflets, and flyers were created based on this feedback. We opted for two different versions: one version for vaccination centres that pointed to the COVID-19 vaccination only and one version for primary care practices that targeted a variety of vaccinations. We implemented most of the feedback but decided to still use gender-neutral language. COVID-19 is now written in lowercase and placed at the bottom of the list to avoid drawing too much attention to that vaccination. Information on data protection is now shortened with reference to a more detailed version on our study’s website. We used the adjusted version for the study’s test roll-out.

#### Evaluation of online survey

Parts of the study’s information and registration form proved to be incomprehensible. The registration questionnaire caused the most difficulties for participants. They understood the study to solely be about COVID-19 vaccine side effects, even though it was stated otherwise in the study’s information. Hence, we began explaining the study’s information on control vaccinations first. Participants requested more specific information regarding the procedure and participation time, e.g. survey completion time in minutes. Focus group participants did not find the survey timelines easy to understand. In the online registration form for COVID-19 vaccinations, we stated: “You will receive a questionnaire 2 weeks after each of the vaccinations and additionally 6 weeks after the second vaccination”. Participants suggested that the graphical timeline from the leaflet also be inserted in the online registration form. We implemented this suggestion and mapped the different vaccinations again in the timeline.

**Fig. 2:**
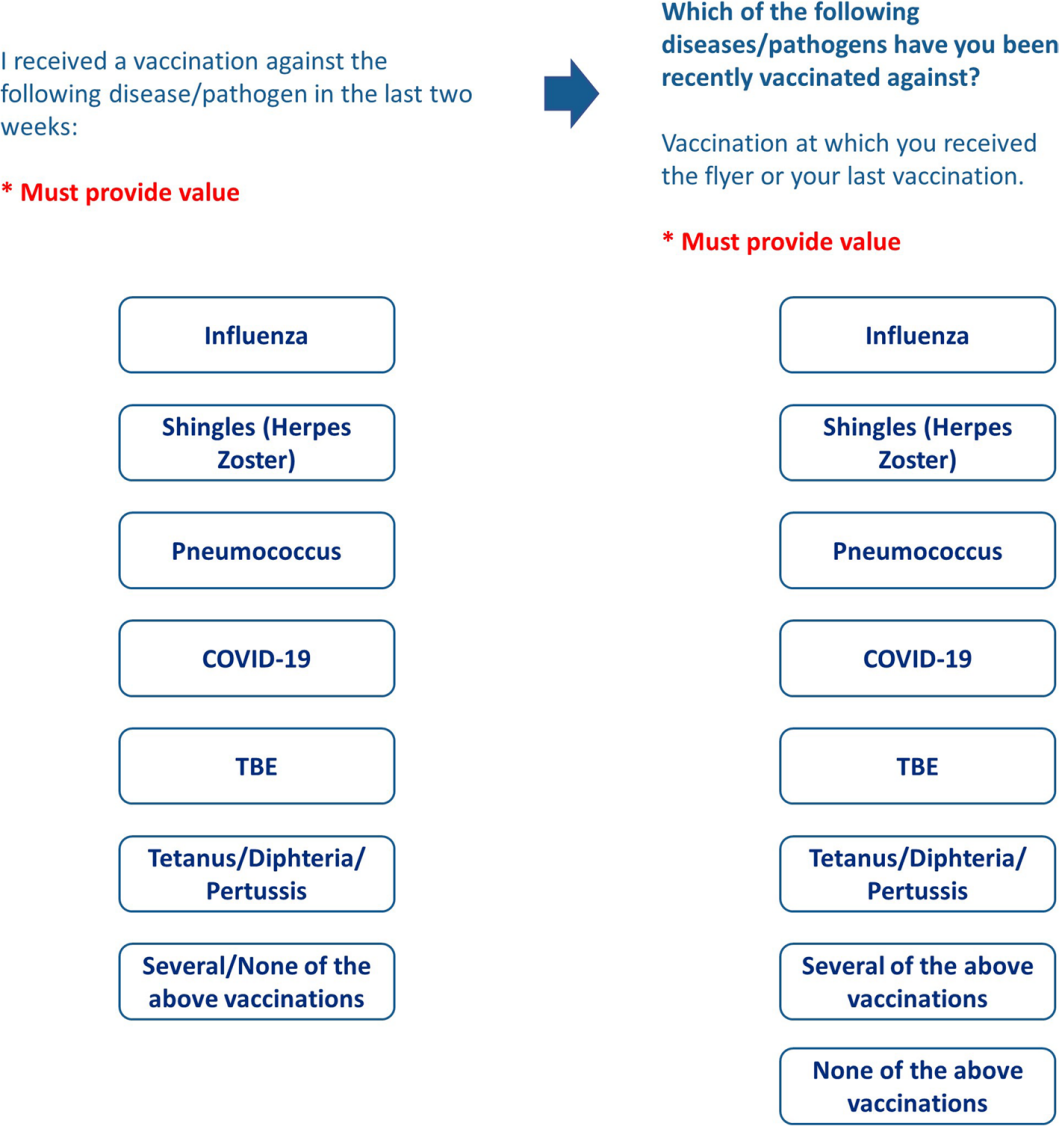
Sample question about vaccination received (before and after conducting focus groups)

When asked about the vaccination they received, they tended to report all the vaccinations they have received in the past (Fig. 2). The reason for this was that several participants thought that their own vaccination experiences from the past were being compared with current ones (e.g. past influenza vaccination vs. present COVID-19 vaccination). We discussed different wordings, but participants self-reported that questionnaires are often not read accurately in general. As a result, we emphasised the signal word *recently* in italics. We also separated the response options “several/none” as this simplified the response decision. Originally, participants were to only be recruited within the 2 weeks after their vaccination. As it later proved more practical to also include participants at a later time, we adapted the time reference in question accordingly.

When asking for the batch number, we first implemented the following question: “Please enter your batch number. *This can be found on the sticker in your vaccination passport or vaccination certificate. Please enter the number without special characters or spaces*”. In order to avoid errors in entering the batch number, participants suggested that a corresponding photo demonstrating where the batch number is located should also be inserted here.

Some buttons on the survey were in English (e.g. “next page”, “submit”) due to the software we used; here, the participants expressed the desire to have these in German. When filling out the survey, it became apparent that not all termination criteria were correctly stored (e.g. termination did not take place, although participants reported no medical contact); no other technical problems occurred. We incorporated the majority of the feedback received. We were not yet able to implement the German system language but are working on a technical solution.

There were only a few comprehension problems when it came to stating *complaints* (*ger*. *Beschwerden*) 2 weeks after vaccination in the follow-up questionnaires. Participants generally discussed what is meant by a *complaint* and whether the term chosen is understandable (alternative suggestion: *anomalies*; *ger. Auffälligkeiten*). We kept the original term. Some suggested to weigh each complaint individually, but this is not relevant for our main study’s objective.

The questionnaire also surveys the consequences of the complaints. To better categorise a potential hospitalisation, the questionnaire further asks whether it was, for example, an “admission as a planned procedure”, an “admission as an emergency”, or “admission with ambulance service”. According to some, these answer options should be explained better. We changed the second answer option to “admission as an emergency by a GP/specialist”.

For the questionnaire given 6 weeks after vaccination, we first asked whether there was a (in- or outpatient) medical consultation. This information was to be reported independently of any association with the vaccination so that potential patterns of vaccine adverse reactions could be identified. According to some participants, we should highlight this more clearly. Consequently, *independent of vaccination* was italicised. We also adapted the study procedure strategy before the test roll-out. Initially, participants were to register within 14 days after their first dose in order to collect data at all survey times. In the test roll-out, participants could register up to 124 days after their first or single dose, in order to receive at least one follow-up survey and not lose too much information due to the vaccination pace.

### Quantitative evaluation of the survey and feasibility

#### Study population

Over the course of 31 days, the feasibility study was tested. During this time, 2199 participants who received either a COVID-19 vaccination (99%) or at least one of the control vaccinations (1%) in the last 124 days registered. Few participants were born before 1951 (7%), and 10.1% were from medium-sized towns. The distribution between rural areas, small towns, and cities was balanced. The majority was employed (69%) and had a university degree (48%).

Most participants received BNT162b2 (BioNTech/Pfizer) or ChadOx1 (AstraZeneca) as their first COVID-19 vaccination (60%, 28%). For their second COVID-19 vaccination, BNT162b2 (BioNTech/Pfizer) and mRNA-1273 (Moderna) were administered most often (68%, 21%). In five cases, appointments to receive a second dose were cancelled. Reasons given for this included pregnancy, SARS-CoV-2 infection after the first dose, intolerance of first dose, or illness on the day of their second dose appointment. Information on the second dose is missing in 446 cases due to missing information from the follow-up surveys. The mean body mass index was 26.2 (*SD* = 5.8). Morbidity assessed with the mSCQ-D was in mean 1.6 (*SD* = 2.4) (Table 3).

**Table 3:** Sociodemographic characteristics, vaccination types, comorbidities, and completion rate for each survey

Baseline characteristics	***n***	%
*N*	**2199**	**100**
Gender		
Female	1233	56
Male	0965	43
Diverse	0001	01
Year of birth		
1991–2003 (approx. 18–30 years)	0419	19
1971–1990 (approx. 31–50 years)	0772	35
1951–1970 (approx. 51–70 years)	0859	39
1933–1950 (approx. 70–88 years)	0149	07
Residence		
Rural area^a^	0618	28
Small town^b^	0642	29
Medium-sized town^c^	0223	10
City^d^	0716	33
Employment		
Employed	1525	69
In education	0228	10
Unemployed	0050	02
Retired	0336	15
Other	0040	02
Not specified	0020	01
Education		
No degree	0002	01
Lower certificate	0055	02
Intermediate certificate	0273	12
Complete apprenticeship	0321	15
High school diploma	0459	21
University degree	1047	48
Not specified	0042	02
Vaccination		
Control group	0024	01
Covid-19	2175	99
**First vaccination COVID (*n*** **= 2175)**		
BNT162b2 (BioNTech/Pfizer)	1313	60
mRNA-1273 (Moderna)	0253	12
ChadOx1 (AstraZeneca)	0609	28
Ad26.COV2.S (Johnson & Johnson)	0000	00
**Second vaccination COVID (*n*** **= 1753)**		
BNT162b2 (BioNTech/Pfizer)	1200	68
mRNA-1273 (Moderna)	0375	21
ChadOx1 (AstraZeneca)	0141	08
Ad26.COV2.S (Johnson & Johnson)	0000	00
Cancelled	0005	01
Not planned	0032	02
*NA*	0422	
**Health status**		
No preexisting diseases	0832	38
Allergies	0598	27
Hypertension	0408	19
Back pain	0331	15
Lung disease	0187	9
Rheumatism/autoimmune disease	0172	8
Depression	0153	7
Osteoarthritis	0142	6
Gastrointestinal disease	0135	6
Heart disease	0117	5
Diabetes	0094	4
Cancer	0072	3
Coagulation problems	0044	2
Kidney disease	0040	2
Liver disease	0034	2
Anaemia	0028	1
	***Mean***	***SD***
mSCQ-D (*n* = 2199)^e^	0001.6	02.4
BMI (*n* = 2176)^f^	0026.2	05.8

^a^Rural area, < 5000. ^b^Small town, 5000 to approx. 20,000. ^c^Medium-sized town, 20,000 to approx. 100,000. ^d^City, 100,000 or more inhabitants. ^e^*mSCQ-D*, modified German version of the Self-Administered Comorbidity Questionnaire consisting of the diseases listed above. ^f^*BMI*, body mass index

More than half of the participants (58%) registered in the week of their first dose or single dose, respectively. In the 6sixth week after the first/single dose, 9% registered, and in the 9th week, 20%.

The estimated recruitment rate in the test roll-out was 7% (2199 participants with 303,050 distributed leaflets). The overall response rate of the surveys was very high with 84%.

#### Evaluation

About 92% of 1993 participants became aware of the study at a vaccination centre. The others heard about it at a physician’s practice, through friends/family, or on the Internet (3%, 3%, and 1%, respectively). About 10% did not fill out an evaluation form after reporting their vaccination side effects.

**Fig. 3:**
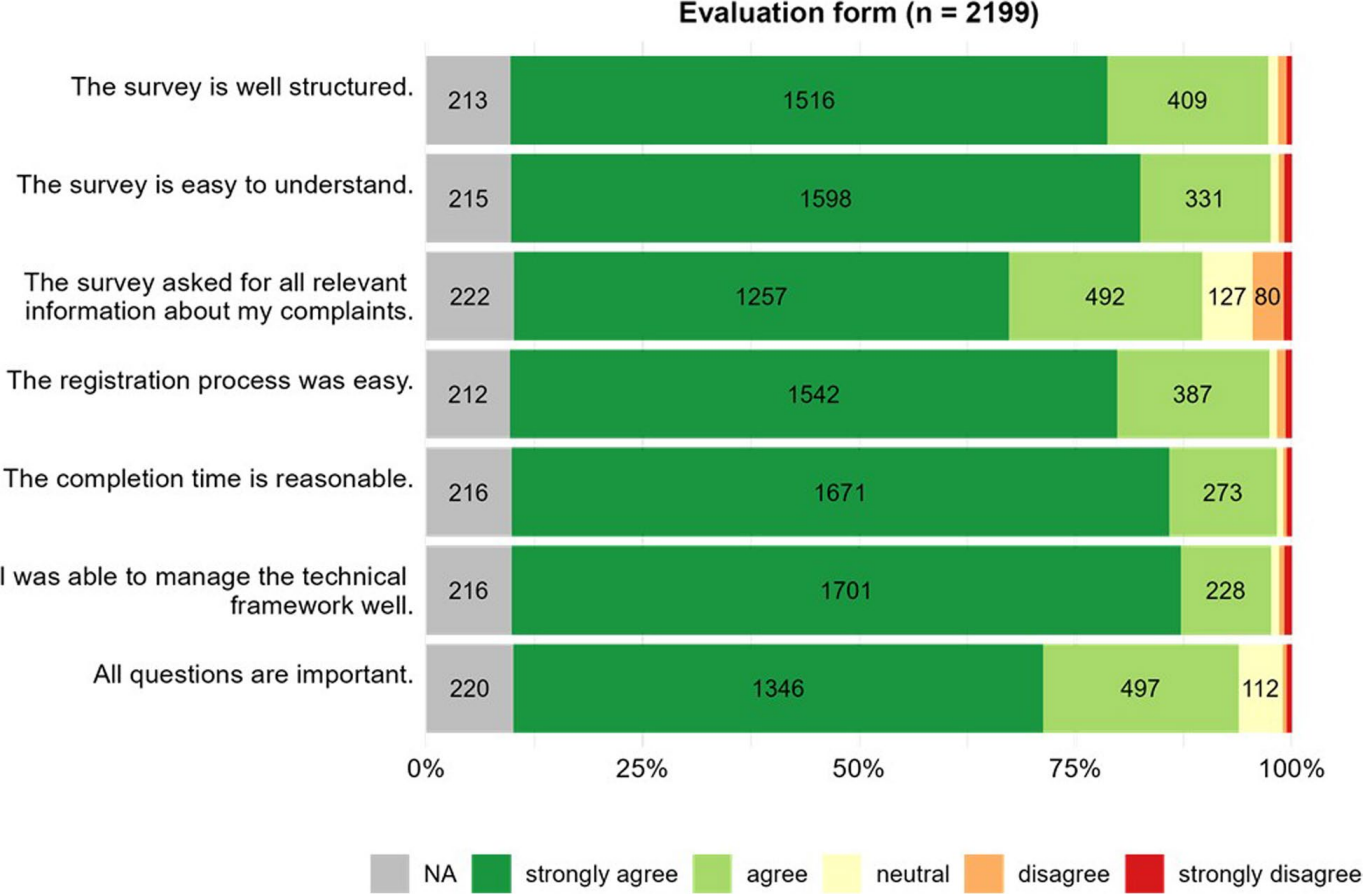
Evaluation form following the online survey. If a participant filled out several evaluation forms, their first submission was considered. Counts of observations in the bars are reported if *n* was > 50

Most participants (strongly) agreed that the registration process was well structured (88%); 88% said it was easy to understand, 80% said that all relevant information was asked, and 84% said that all questions were important. Regarding the study’s process, 88% of participants (strongly) agreed that the registration process was easy, the completion time was reasonable, and that the technical framework was straightforward (Fig. 3).

#### Optional feedback in the comment section

Suggestions in terms of organisation, technical implementation, and content were made. After the registration, participants expected to receive an invitation link or a confirmation email immediately or shortly after their vaccination. For confidentiality and technical reasons, we did not send confirmation emails, but information was added during the feasibility study at the end of registration. The fact that the REDCap system language is English resulted in some participants receiving a poor, automatic German translation of the survey in certain Internet browsers, although the survey was developed in German. A software update after the test roll-out was able to solve this problem.

Concerning the content, some participants wished for a more differentiated list of symptoms and options that enabled them to specify the duration and onset of their symptoms. The item asking for personal perception of the link between vaccination and the complaints was difficult for some. A few participants suggested adding the answer option “I don't know”.

As some participants shared one single email address or completed registration twice, we implemented a disclaimer at the beginning of each survey with their personal information regarding type and date of vaccine, year of birth, and gender. With this information, participants could also give us feedback on whether the information they entered was incorrect and/or could register again.

### Qualitative evaluation of the feasibility

#### Study population

Eleven primary care practices and two large-scale vaccination centres distributed leaflets to patients following their receival of vaccinations. In total, three GPs and two vaccination centres staff members took part in an interview.

#### Evaluation

We sent out a total of 1600 leaflets to primary care practices and 28,750 leaflets to the vaccination centres. The leaflets were distributed at different times, e.g. directly after the vaccination by physicians or in the waiting area. Participating partners said that the study is easy to integrate into their regular workflow. Everyone preferred the small leaflets over flyers and posters. Posters were not desired due to lack of space, especially from GPs. One GP reported a greater level of interest from patients when leaflets were distributed by physicians instead of medical assistants. Physicians reported mostly positive reactions from vaccinated persons. Some reported little additional work due to questions from patients, but overall, the questions did not interfere with their tight schedules. According to participants, they distributed all leaflets. Physicians suggested recruiting more GPs through mailing lists and offering compensation.

Nine GP practices and both vaccination centres expressed willingness to participate in the main project.

## Discussion

Our study showed that identifying and documenting adverse reactions following vaccinations by means of an online survey are, indeed, feasible. The qualitative evaluation of the recruiting materials led to some adjustments, e.g. adjusting the wording or designing two different forms of recruiting material for vaccination centres and primary care practices. After evaluating the online survey, self-checks (vaccination, gender, age) and feedback were implemented, so that participants are informed, for example that the survey will not be sent out immediately after registration. The greatest challenge laid in recruiting individuals after a control vaccination.

The focus groups showed how relevant testing the recruitment material is in reducing misunderstandings. Despite adapting the study’s material according to the focus groups’ feedback, misinterpretations still occurred during the test roll-out. COVID-19 currently holds a significant amount of public attention. This made it easy to recruit patients after their receival of a COVID-19 vaccination but led to difficulties in reaching the target number of participants for the control group. Additionally, few other vaccines were administered at that time. The evaluation of the recruiting materials and the survey showed that it is important to shift the current general focus away from COVID-19 and onto all vaccinations of the target group. It also showed that reading instructions carefully is often problematic for participants in studies [17]. Therefore, to successfully shift focus, it is extremely important to use unambiguous and clear formulations.

We aimed to have 50 participants in each group for the feasibility study. We overachieved this for the COVID-19 vaccination group, while barely anyone participated in the control group. Since recruiting participants for the control group proved to be more difficult than anticipated, greater emphasis will need to be placed on the recruitment strategy for this group during the main study. Possible solutions for the main study are recruiting more primary care practices and company physicians, as well as announcements through newspapers and the radio, e.g. right before the influenza vaccination season.

The administrative burden of any study is a barrier to participation [18]. Successfully implementing and integrating this study into existing processes and procedures of healthcare institutions might succeed by imposing as little effort as possible on the participants. Interviews with the participating GPs and heads of vaccination centres showed that the study easily fits into their workflows. Although GP practices participated without compensation in the feasibility study, we intend to monetarily acknowledge their cooperation in the main study; the reason for this is that, in the main study, the duration of the data collection will be longer, and a monetary reward might motivate recruiting for the control group. Another way to motivate physicians might be by providing them with a better explanation as to why the control group is methodologically necessary for the main question of the study (for which recruiting participants turned out to be easy).

The recruiting process also showed that only a small proportion of the participants in the test roll-out were older than 70 years of age; this was probably due to the vaccine recommendations and prioritisation at that time [19]. Using an online survey might also be another reason for lack of participation, as older people are less confident in their use of emails and the Internet [20, 21]. However, other studies show no group differences in age or gender when comparing different recruitment strategies [22]. In these studies, the online option was more likely to be chosen by chosen by higher-educated participants.

Due to the fluctuations in COVID-19 vaccination recommendations, flexibility in the study procedure proved to be important. Recommendations like the heterologous vaccination regimen or changes in intervals between the two doses had to be taken into consideration in the survey [1–3, 19, 23–26]. The survey question on the COVID-19 vaccination brand name and batch number was therefore adapted and repeatedly asked in the follow-up surveys. Automatically sent invitations and the survey itself had to be customisable. Initially, it was planned to assess data from vaccinated persons after their first or single dose of COVID-19, respectively. However, through this procedure, a lot of information would have been lost, especially as our study started when 3,436,080 doses had already been administered to about 19.5% of the Bavarian population [27]. We therefore adapted the strategy before the test roll-out so that participants could register up to 124 days after their first or single dose, in order to receive at least one follow-up survey. Data on adverse reactions, even if only assessed after the second dose, are a valuable complement to real-world evidence. For example, several weeks after recommendation of the heterologous vaccination regimen in Germany, there is still only little real-world evidence on the safety of this regimen [28–30].

### Limitations

Due to the urgent implementation and limited financial resources of this study, other data collection methods such as postal or telephone survey were not tested. Because of the time constraints caused by COVID-19 vaccinations already starting in practices, we could not lead more interviews with GPs. Data saturation could not be achieved because regular adjustments would have required continuous focus groups and interviews. Because of COVID-19 vaccination prioritisation of certain population groups, the study population of the test roll-out may not be representative of the entire population in age, sex, or educational background. In the progress of the vaccination campaign, diverse groups of people will be targeted over time. During the feasibility study, participants probably had higher grades of multimorbidity and were older or were predominantly medical personnel. These biases can, lamentably, neither be diminished now nor in the main study.

## Conclusion

Testing materials and surveys before implementing a study is essential. Collaborating with health professionals, vaccination centres, and physicians’ practices and incorporating their suggestions help not only in reaching many patients quickly but also in ensuring the practicality of the study’s procedures. For a successful implementation of the main study during the pandemic, flexibly adapting the study’s organisation according to changing structures and requirements proved to be essential. Recruitment of the control group will be the greatest challenge for the main study.

## Supplementary Information

**Additional file 1: Supplementary material 1.** Focus Groups – Interview guide. 2. Follow-up interviews with vaccination centres and GP practices: Interview guide. 3. Quantitative Evaluation of the Online-Survey. 4. Quantitative Online Survey.

## Data Availability

The data that support the findings of this study are available from the corresponding author NZ upon reasonable request.

## Abbreviations

CoVaKo: Corona-Vakzin-Konsortium
COVID-19: Coronavirus disease 2019
GP: General Physicians
