# Supplementary material for: Active monitoring of adverse reactions following COVID-19 and other vaccinations: a feasibility study as part of the CoVaKo project
Source: Pilot Feasibility Stud. 2022 Jul 2;8:134. doi: 10.1186/s40814-022-01088-y (PMC9250275; doi:10.1186/s40814-022-01088-y)
Supplement: Supplementary file 1 — Additional file 1: Supplementary material 1. Focus Groups – Interview guide. 2. Follow-up interviews with vaccination centres and GP practices: Interview guide. 3. Quantitative Evaluation of the Online-Survey. 4. Quantitative Online Survey. [file 40814_2022_1088_MOESM1_ESM.docx]

## Supplementary Material

### Focus Groups – Interview guide

**Survey**

Procedure: Each question of the survey will be discussed individually.

- How is the comprehensibility?
  - Complicated words?
- How would you answer the question?
- Are there too many/too few answer options?

**Recruitment material**

- How was your first impression when you saw the poster/flyer/leaflet?
- Which poster/flyer/leaflet do you like best? Please explain your choice.
  - What would you change?
- What do you think of the design?
  - Colors
  - Pictures? Which ones do you like best?
- How do you rate the comprehensibility?
  - Long/complex sentences?
  - Complicated words?
  - On the posters/flyers/leaflets you find different terminology (COVID-19, Corona, Sars-CoV-2): Which do you think is most common/easiest to understand?
  - Flyer: How much information would you like here on data protection?
- If you imagine you go to the vaccination centre/your GP and see this poster/flyer/leaflet, would you participate in the study?
- Do you feel you have a choice to participate or refuse?
  - Unbalanced presentation?
- How would you register for the study? (QR code or link?)
  - Have you ever used a QR code before?
  - Have you tried to scan this QR code?

**Case vignettes**

Each of you has now received a short case vignette in the chat. We would like for you to fill out the survey. Try to keep your case in mind when filling out the survey. If you have questions or problems that prevent you from continuing, you can ask us in between. If you can continue, note your problems and we will talk about them afterwards.

**Case 1**

Please fill out the registration and the first survey: Today you had your first influenza vaccination. You can choose the vaccine. After the vaccination you felt quite dizzy and collapsed. This was the first time ever that you experienced a reaction after a vaccination. You did not have COVID-19. You can choose two health issues and decide freely on the following questions. Please evaluate the survey in the end.

Please fill out the second survey: You went to the doctor but did not need to go the hospital. You did not have another vaccination. You were not sick with COVID-19. Choose two health issues that have already existed before the vaccination.

**Case 2**

Please fill out the registration and the first survey: Last week you had your first herpes zoster vaccination. You can choose the vaccine. You can choose three health issues. With one health issue, you check that you needed medical attention but than want to change your answer to “neither of them”. You were not sick with COVID-19. After the vaccination you felt nauseous and experienced shortness of breath. You know these symptoms from previous vaccinations. You are free to choose the consequences. Please evaluate the survey in the end.

Please fill out the second survey: You went to the doctor and were admitted to the hospital. You also had a vaccination against pneumococcal vaccination, but were not sick with COVID-19. Choose three health issues that have already existed before the vaccination.

**Case 3**

Please fill out the registration and the first survey: Today you had your second TBE vaccination. You can choose the vaccine. You have no known health issues, no COVID-19 disease. You experienced no vaccine reactions, this is congruent with prior vaccinations. Please evaluate the survey in the end.

Please fill out the second survey: You did not need any medical attention.

**Case 4**

Please fill out the registration and the first survey: Today you had your first COVID-19 vaccination (Astra Zeneca). Your next appointment: 05. Jun 2021. You also had a vaccination against pneumococcal vaccination, but were not sick with COVID-19. You have no health issues or vaccine reactions. So far, you have never experienced any problems after a vaccination.

Please evaluate the survey in the end.

Please fill out the second survey: You needed to go to the doctor, but were not admitted to the hospital. You were not sick with COVID-19. Choose two health issues that you did not have before the vaccination.

**Case 5**

Please fill out the registration and the first survey: Today you had your first COVID-19 vaccination (Moderna). You do not know when you will get your second dose. You can choose 3 health issues and 3 vaccine reactions and decide freely on the following questions. Please evaluate the survey in the end.

Please fill out the second survey: You did not need any medical attention.

- Which device (mobile phone, tablet and laptop) did you use?
  - How was the readability?
- Which browser did you use?
- How was the visual design?
- Did you any problems?
  - Did you have any technical errors?

### Follow-up interviews with vaccination centres and GP practices: Interview guide

| Date |  |
| --- | --- |
| Institution | - Vaccination centre - GP practice |
| Location | - City (≥ 100.000 inhabitants) - Medium-sized town (20.000 – approx. 100.000 inhabitants) - Small town (< 20.000 inhabitants) - Rural area (< 5.000 inhabitants) |
| Interview with |  |

How was the distribution of the recruitment material?

- Who distributed the leaflets?
- When was the leaflet handed out?
- Did you or would you put up posters in the practice/vaccination centre?
- What was the level of patient interest?

How long did the supply of materials last?

How do you estimate the effort required?

- Did patients have any more/additional questions? If so, what did they ask?
- Can we make any adjustments to the material?

How was the contact with the Institute of General Medicine? How should the contact be organised in the future?

- - Frequency of contact?
  - Availability in case of queries?

Could you imagine participating in the main study?

How could we recruit more practices/vaccination centres for the study? Do you have any suggestions for motivating other colleagues?

### Quantitative Evaluation of the Online-Survey

Below you have the opportunity to evaluate the study (process). We appreciate your participation in order to further improve the study.

This will take about 1-3 minutes.

**PROG**: Matrix fields.

1. The registration process is easy.
2. The questionnaire is well structured.
3. The questionnaire is easy to understand.
4. The questionnaire has asked all relevant information about my complaints.
5. All questions are important.
6. The completion time is reasonable.
7. I found the technical framework easy to handle.

**PROG**: Matrix fields. Allow only one answer option.

| Strongly disagree | ⭘ |
| --- | --- |
| Rather disagree | ⭘ |
| Neither | ⭘ |
| Rather agree | ⭘ |
| Strongly agree | ⭘ |

1. How did you learn about the study?

**PROG**: Allow only one answer option.

| Vaccination centre | ⭘ |
| --- | --- |
| Medical practice | ⭘ |
| Friends/Acquaintances/Family | ⭘ |
| Internet | ⭘ |
| Other | ⭘ |

1. Did you answer all questions thoroughly?

**PROG**: Allow only one answer option.

| I answered all questions thoroughly. | ⭘ |
| --- | --- |
| Sometimes I clicked on anything because I was unmotivated or just didn't know my way around. | ⭘ |
| I often clicked on anything because I wanted to finish quickly. | ⭘ |

1. Is there anything else you would like to say about this survey or for a better understanding of your answers?

Did you notice any problems during the survey? Were the questions not clear at some point or did you feel uncomfortable answering them? Please briefly write down a few key points.

**PROG**: Free text field.

You have reached the end of the questionnaire. Thank you very much for your participation!

Please click on “*Submit/Save*” to finish the survey.

### Quantitative Online Survey

**Registration**

**The CoVaKo Project**

Thank you for taking part in our survey.

What is the study about? Side effects of various vaccinations are to be recorded.

Who is conducting this study? The Institute of General Practice of the Universitätsklinikum Erlangen.

Who can participate? People aged 18 or older who have received a vaccination (e.g. pneumococcus, TBE, COVID-19 etc.) in the last few weeks.

How does the study work?

- Registration with your e-mail address and personal information (takes about 3 minutes)
- Survey at a maximum of 5 time points after vaccination (takes about 5 minutes)

Please have your vaccination certificate ready for registration.

Please register no earlier than the date of vaccination.

If you would like to participate in the study, please click on *Next Page*.

**Data protection**

Important information on data protection:

For the duration of the study, we need your e-mail address in order to send you the link to each questionnaire. Your e-mail address will be deleted immediately after the last survey or after a premature termination. Your personal data will only be processed anonymously in the evaluation (i.e. it is not possible to draw any conclusions about your person).

Reports on severe vaccine reactions are also transmitted to the Paul Ehrlich Institute in anonymised form. The Paul Ehrlich Institute is legally obliged to collect and evaluate all reported suspected adverse reactions and to forward them to the European adverse reaction database.

**Right of withdrawal:**

Participation in the study is voluntary. Until your data has been completely anonymised, you have the right to obtain information (including a copy free of charge) about your personal data at any time and to demand its correction or deletion without giving reasons. This will not result in any disadvantages for you.

Detailed information on the study and the detailed data protection declaration can be downloaded here: Data protection

1. I hereby agree to the privacy policy.

**PROG**: Allow only one response option. Must click on "Next" to confirm (not programmable otherwise).

| Yes | ⭘ | Continue to the next question |
| --- | --- | --- |
| No, I would like to end my participation. | ⭘ | **PROG**: **END** |

**Personal Details**

1. Please enter your e-mail address:

**PROG**: Free text field that complies with the format “xy@example.de”.

1. Please enter your year of birth:

**PROG**: Free text field, which must contain four digits (with a minimum of 1900 and a maximum 2003).

1. Please indicate your gender:

**PROG**: Allow only single-choice answers.

| Male | ⭘ |
| --- | --- |
| Female | ⭘ |
| Diverse | ⭘ |

1. Please state your height in cm:

**PROG**: Free text field. No decimal places allowed.

1. Please state your weight in kg. Indicate in whole numbers:

**PROG**: Free text field. No decimal places allowed.

1. Please select the highest level of education you have achieved so far.

**PROG**: Allow only single-choice answers.

| No degree | ⭘ |
| --- | --- |
| Lower certificate | ⭘ |
| Intermediate certificate | ⭘ |
| Completed apprenticeship | ⭘ |
| High school diploma | ⭘ |
| University degree | ⭘ |
| Not specified | ⭘ |

1. Which employment situation applies best to you? Please note that employment means any paid activity or activity that is associated with an income.

**PROG**: Allow only single-choice answers.

| Employed | ⭘ |
| --- | --- |
| In education | ⭘ |
| Unemployed | ⭘ |
| Retired | ⭘ |
| Other | ⭘ |
| Not specified | ⭘ |

1. How many inhabitants does your place of residence count?

**PROG**: Allow only single-choice answers.

| Below 5.000 (rural area) | ⭘ |
| --- | --- |
| 5.000 to approx. 20.000 (small town) | ⭘ |
| 20.000 to approx. 100.000 (medium-sized town) | ⭘ |
| 100.000 or more (city) | ⭘ |

**Vaccination**

For the following questions you will need your vaccination certificate.

1. Against which of the following diseases/pathogens have you been vaccinated most recently?

**PROG**: Allow only single-choice answers.

| Influenza | ⭘ |
| --- | --- |
| Shingles | ⭘ |
| Pneumococcus | ⭘ |
| COVID-19 | ⭘ |
| TBE | ⭘ |
| Tetanus (and/or diphtheria/pertussis/poliomyelitis) | ⭘ |
| Several of the above | ⭘ |
| None of the above | ⭘ **PROG: END** |

1. Against which of the following diseases/pathogens have you been vaccinated? Multiple-choice answers are possible.

**PROG**: Allow multiple-choice answers. Only appears if "several" was selected in question 10.

| Influenza | ⭘ |
| --- | --- |
| Shingles | ⭘ |
| Pneumococcus | ⭘ |
| COVID-19 | ⭘ |
| TBE | ⭘ |
| Tetanus (and/or diphtheria/pertussis/poliomyelitis) | ⭘ |

In the following you will be asked for the name of the vaccine. You will find this on the label in your vaccination certificate.

1. You received the following influenza vaccine:

**PROG**: Allow only single-choice answers. Only appears if “influenza” was selected in question 10. Free text field opens when “other” is checked.

| Afluria Tetra 2020/2021 | ⭘ |
| --- | --- |
| Flucelvax Tetra 2020/2021 | ⭘ |
| Fluenz Tetra 2020/2021 | ⭘ |
| Influsplit Tetra 2020/2021 | ⭘ |
| Influvac Tetra 2020/2021 | ⭘ |
| Vaxigrip Tetra 2020/2021 | ⭘ |
| Xanaflu Tetra 2020/2021 | ⭘ |
| I do not know | ⭘ |
| Other | ⭘ |

1. You received the following shingles vaccine:

**PROG**: Allow only single-choice answers. Appears only if "shingles" was selected in question 10.

| Shingrix | ⭘ |
| --- | --- |
| Zostavax | ⭘ |
| I do not know | ⭘ |

1. You received the following pneumococcal vaccine:

**PROG**: Allow only single-choice answers. Appears only if "pneumococcus" was selected in question 10.

| Pneumococcal Polysaccharide Vaccine 23 | ⭘ |
| --- | --- |
| Pneumovax 23 | ⭘ |
| Prevenar 13 | ⭘ |
| I do not know | ⭘ |

1. You received the following COVID-19 vaccine:

**PROG**: Allow only single-choice answers. Appears only if "COVID-19" was selected in question 10.

| BNT162b2 (BioNTech/Pfizer) | ⭘ |
| --- | --- |
| mRNA-1273 (Moderna) | ⭘ |
| ChadOx1 (AstraZeneca) | ⭘ |
| Ad26.COV2.S (Johnson & Johnson) | ⭘ |

1. You received the following TBE vaccine:

**PROG**: Allow only single-choice answers. Appears only if "TBE" was selected in question 10.

| Encepur | ⭘ |
| --- | --- |
| FSME IMMUN | ⭘ |
| I do not know | ⭘ |

1. You received the following tetanus (and/or diphtheria/pertussis/poliomyelitis) vaccine:

**PROG**: Allow only single-choice answers. Appears only if "tetanus” was selected in question 10.

| Boostrix (TdPa/TdPa-IPV) | ⭘ |
| --- | --- |
| Covaxis (TdPa) | ⭘ |
| Infanrix (DTPa) | ⭘ |
| Repevax (TdPa-IPV) | ⭘ |
| Revaxis (Td-IPV) | ⭘ |
| Td-IMMUN (Td) | ⭘ |
| Td Impfstoff Merieux® (Td) | ⭘ |
| Td-pur (Td) | ⭘ |
| TdaP-IMMUN (TdPa) | ⭘ |
| Tetanol pur | ⭘ |
| Tetanus-Impfstoff Mérieux® | ⭘ |
| Tetravac | ⭘ |
| I do not know | ⭘ |

1. When did you receive this/these vaccination(s)?

**PROG**: Free text field. Must comply with the format XX.XX.XXXX. Only appears if "COVID-19" and "none" was not selected in question 10.

1. Have you ever been vaccinated against _______ before?

**PROG**: Allow only single-choice answers. The vaccination selected in question 10 appears in the placeholder unless "more than one" was checked.

| Yes | ⭘ |
| --- | --- |
| No | ⭘ |
| I do not know | ⭘ |

1. Have you ever been vaccinated against _______ before?

**PROG**: The vaccinations selected in question 11 appears in the placeholder. One question appears for each selection in question 11.

| Yes | ⭘ |
| --- | --- |
| No | ⭘ |
| I do not know | ⭘ |

1. When did you receive your first vaccination?

**PROG**: Free text field, must comply with the format XX.XX.XXXX. Only appears if "COVID-19" was selected in question 10.

1. When did you receive or when will you receive the 2nd vaccination?

**PROG**: Free text field, must comply with the format XX.XX.XXXX. Only appears if "COVID-19" was selected in question 10.

1. You are probably going to receive the following COVID-19 vaccine (2nd vaccination):

**PROG**: Allow only single-choice answers. Only appears if "COVID-19" was selected in question 10.

| BNT162b2 (BioNTech/Pfizer) | ⭘ |
| --- | --- |
| mRNA-1273 (Moderna) | ⭘ |
| ChadOx1 (AstraZeneca) | ⭘ |
| Ad26.COV2.S (Johnson & Johnson) | ⭘ |
| Not sure yet | ⭘ |

The batch number can be found on the label in your vaccination certificate. Please enter the number without special characters or spaces.

1. Please enter your batch number.

**PROG**: Free text field. Only appears if "none" or "several" was not selected in question 10.

1. Please enter your batch numbers: Enter batch numbers separated by commas.

**PROG**: Free text field. Only appears if "several" was selected in question 10.

1. Did you receive another vaccination in the 8 weeks before your vaccination on _______?

**PROG**: Allow only single-choice answers. Date entered in question 18 appears in the placeholder. Only appears if "COVID-19", "none" or "several" was not selected in question 10.

| Yes, against another disease | ⭘ |
| --- | --- |
| Yes, against the same disease | ⭘ |
| No | ⭘ |
| I do not know | ⭘ **PROG**: **END** |

1. Did you receive another vaccination in the 8 weeks before your vaccination on _______?

**PROG**: Allow only single-choice answers. Date entered in question 21 appears in the placeholder. Only appears if "COVID-19” was selected in question 10.

| Yes | ⭘ |
| --- | --- |
| No | ⭘ |
| I do not know | ⭘ |

1. Against which of the following diseases/pathogens have you been vaccinated before? Multiple-choice answers are possible.

**PROG**: Only appears if "Yes, against another disease" was selected in question 26. The vaccination selected in question 10 is not included in the possible answers in each case. If "several" was not selected in question 10, only single-choice answers are allowed. If "several" was selected in question 10, multiple-choice answers are allowed.

| Influenza | ⭘ |
| --- | --- |
| Shingles | ⭘ |
| Pneumococcus | ⭘ |
| COVID-19 | ⭘ |
| TBE | ⭘ |
| Tetanus (and/or diphtheria/pertussis/poliomyelitis) | ⭘ |
| None of the above | ⭘ |

**Morbidity**

1. Please select the general health problems you suffer from. This does not include vaccination side effects. Please check the appropriate box. Multiple-choice answers are possible.

**PROG**: Multiple-choice answers are allowed.

| Heart disease | ⭘ |
| --- | --- |
| High blood pressure | ⭘ |
| Lung disease | ⭘ |
| Diabetes | ⭘ |
| Gastrointestinal problems | ⭘ |
| Kidney disease | ⭘ |
| Liver disease | ⭘ |
| Anemia | ⭘ |
| Coagulation disorders | ⭘ |
| Cancer | ⭘ |
| Depression | ⭘ |
| Ostheoarthritis | ⭘ |
| Back pain | ⭘ |
| Rheumatoide arthritis (other autoimmune disease) | ⭘ |
| Allergies | ⭘ |
| None pre-existing diseases | ⭘ |

1. Are you receiving treatment for the symptoms, taking medication or are you restricted in your daily activities?

**PROG**: Multiple-choice answers are allowed, except for the option “Neither”. Only the health problems selected in question 29 will appear.

| Treatment/Medication | ⭘ |
| --- | --- |
| Restriction in daily life | ⭘ |
| Neither | ⭘ |

**End after successful registration**

You have now reached the end of the registration process.

Thank you very much for your support.

You will receive a questionnaire by e-mail approximately 14 days after your vaccination.

1. If you would like to add anything, please use the following empty field for your comments.

**PROG**: Free text field.

Please check “*Submit*” to finish the survey.

**End if registration is cancelled**

**PROG**: Only appears if the privacy policy has been rejected or if "none" has been selected in question 10.

Sorry, you do not meet the criteria for the survey.

Thank you for your support.

1. If you would like to add anything, please use the following empty field for your comments.

**PROG**: Free text field.

Please click on “*Submit*” to complete the survey.

**Short-term survey 1a**

Thank you for participating in our study.

Mark the answer that most closely matches your assessment. There is no right or wrong.

Please click on "*Next Page*" to start the survey.

**Additional Vaccination**

1. Did you receive another vaccination since the vaccination against _______?

**PROG**: Allow only single-choice answers. The vaccination selected in the registration in question 10 appears in the placeholder. Only appears if "several" was not selected in the registration in question 10.

| Yes | ⭘ |
| --- | --- |
| No | ⭘ |

1. Did you receive another vaccination since the _______ vaccinations?

**PROG**: Allow only single-choice answers. The vaccinations selected in the registration in question 11 appear in the placeholder. Only appears if "several" was selected in the registration in question 10.

| Yes | ⭘ |
| --- | --- |
| No | ⭘ |

1. You received the following vaccination(s) afterwards. Multiple-choice answers are possible.

**PROG**: Allow multiple response options. Only appears if "Yes" was selected in question 1 or 2.

| Influenza | ⭘ |
| --- | --- |
| Shingles | ⭘ |
| Pneumococcus | ⭘ |
| COVID-19 | ⭘ |
| TBE | ⭘ |
| Tetanus (and/or diphtheria/pertussis/poliomyelitis) | ⭘ |
| None of the above | ⭘ |

1. Have you been diagnosed with COVID-19 disease since your vaccination at _______?

**PROG**: Allow only single-choice answers. The date entered in the registration in question 18 or 21 appears in the placeholder.

| Yes | ⭘ |
| --- | --- |
| No | ⭘ |

1. Have there been any changes concerning your 2nd vaccination? Multiple-choice answers are possible.

**PROG**: Multiple answer choices allowed. Only appears if "COVID-19" was selected in the registration in question 10.

| No changes | ⭘ |
| --- | --- |
| Yes, appointment cancelled | ⭘ |
| Yes, change of active substance | ⭘ |
| Other | ⭘ |

**PROG**: Free text field. Only appears if “Other” is selected in question 5.

1. Please enter the date for your 2nd vaccination.

**PROG**: Free text field. Only appears if "COVID-19" was selected in question 1 in the registration.

1. What was the reason for cancelling the appointment? Multiple-choice answers are possible.

**PROG**: Multiple answer options allowed. Appears only if "Yes, appointment cancelled" was selected in question 5.

| COVID-19 infection after vaccination | ⭘ |
| --- | --- |
| Intolerance to vaccination | ⭘ |
| Other | ⭘ |

**PROG**: Free text field. Only appears if “Other” is selected in question 7.

1. You are ~~probably going~~ to receive the following COVID-19 vaccine. You will find the name of the vaccine on the label in your vaccination certificate:

**PROG**: Allow only one response option. Only appears if "COVID-19" was selected in the registration in question 10.

| BNT162b2 (BioNTech/Pfizer) | ⭘ |
| --- | --- |
| mRNA-1273 (Moderna) | ⭘ |
| ChadOx1 (AstraZeneca) | ⭘ |
| Ad26.COV2.S (Johnson & Johnson) | ⭘ |
| Not sure yet | ⭘ |

**Type of complaints**

1. Did you experience any complaints since the ______ vaccination? Please also record complaints that you do not attribute to the vaccination.

**PROG**: Allow only single-choice answers. The placeholder shows the vaccination(s) selected in the registration in question 10 or 11.

| Yes | ⭘ |
| --- | --- |
| No | ⭘ **PROG**: **END** |

1. Which of the following complaints did you experience? Multiple-choice answers are possible.

**PROG**: Allow multiple-choice answers.

| Complaints at the injection site (e.g. swelling, redness, pain) | ⭘ |
| --- | --- |
| Restricted movement of the arm | ⭘ |
| Pus collection and/or abscess | ⭘ |
| Tiredness and/or fatigue | ⭘ |
| Fever > 38.0°C and/or chills | ⭘ |
| Nausea and/or vomiting | ⭘ |
| Allergic reaction (e.g. skin rash, facial swelling) | ⭘ |
| Shortness of breath | ⭘ |
| Headache | ⭘ |
| Sensory disturbance and/or numbness | ⭘ |
| Circulatory collapse | ⭘ |
| Dizziness | ⭘ |
| Seizure | ⭘ |
| Muscle or joint pain | ⭘ |
| Blood clotting disorder (e.g. thrombosis, embolism) | ⭘ |
| Other | ⭘ |

1. Which other complaints have you experienced? Please enter your other complaints here:

**PROG**: Free text field. Only appears if "Other" was selected in question 10.

1. Have you ever had an allergic reaction to a vaccine? (e.g. skin rash, shortness of breath, swelling in the throat or face, loss of consciousness).

**PROG**: Allow only single-choice answers. Only appears if "Allergic reaction", "Shortness of breath" or "Circulatory collapse" was selected in question 10.

| Yes | ⭘ |
| --- | --- |
| No | ⭘ |
| I do not know | ⭘ |

1. Has this been your first seizure?

**PROG**: Allow only single-choice answers. Only appears if “Seizure” was selected in question 10.

| Yes | ⭘ |
| --- | --- |
| No | ⭘ |
| I do not know | ⭘ |

**Consequences**

1. Which consequences did your health complaints have? Multiple-choice answers are possible.

**PROG**: Allow multiple-choice answers. Only appears if at least one answer was selected in question 10. A separate question appears for each answer selected in question 10.

| No consequences | ⭘ |
| --- | --- |
| Medication intake | ⭘ |
| Sick-leave | ⭘ |
| Consultation (outpatient) | ⭘ |
| Hospital (in-patient/overnight) | ⭘ |
| Hospital (out-patient/hourly) | ⭘ |

Please provide some information about your hospital stay.

1. How many times have you been to the hospital?

**PROG**: Allow only single-choice answers. Only appears if "Hospital (in-patient/overnight)" and/or “Hospital (out-patient/hourly)” was selected at least once in question 14.

| Once | ⭘ |
| --- | --- |
| Twice | ⭘ |
| 3 times | ⭘ |
| 4 times or more | ⭘ |

1. How long was your (longest) inpatient hospital stay in days?

**PROG**: Allow only single-choice answers. Only appears if "Hospital (inpatient/overnight)" was selected at least once in question 14.

| 1 day | ⭘ |
| --- | --- |
| 2 days | ⭘ |
| 3 days | ⭘ |
| 4 days | ⭘ |
| 5 days | ⭘ |
| 6 days | ⭘ |
| 7 days | ⭘ |
| 8 days or more | ⭘ |

1. How did the admission(s) to the hospital take place?

**PROG**: If "Once" was selected in question 15, Allow only single-choice answers. If "Twice" or more was selected in question 15, allow multiple answer choices. Only appears if "Hospital (in-patient/overnight)" and/or “Hospital (out-patient/hourly)” was selected in question 14.

| Referral as a planned intervention by your general practitioner or specialist | ⭘ |
| --- | --- |
| Referral as an emergency by your general practitioner or specialist | ⭘ |
| Referral by a doctor on call | ⭘ |
| Transport by the ambulance service | ⭘ |
| Own presentation in the emergency room | ⭘ |

**Perception**

Please tell us how you felt about your health complaints.

**PROG**: Matrix fields.

1. I suspect a connection between my health complaints and the vaccination.
2. I feel/felt adversely affected by the health complaints.
3. My health complaints will have long-term consequences for me.
4. My health complaints are comparable to previous vaccinations.

**PROG**: Matrix fields. Allow only single-choice answers per question. Matrix only appears if at least one complaint has been selected in question 10.

| Strongly disagree | ⭘ |
| --- | --- |
| Disagree | ⭘ |
| Agree | ⭘ |
| Strongly agree | ⭘ |

1. With which complaints do you suspect a connection with the vaccination? Multiple-choice answers are possible.

**PROG**: Allow multiple-choice answers. Each complaint selected in question 10 appears and can be checked on if a connection with vaccination is suspected.

**End of short-term survey 1a**

You have now reached the end of the survey.

Thank you very much for your responses.

Please click on “Submit” to end the survey.

**Short-term survey 1b**

Thank you for taking part.

Mark the answer that most closely matches your assessment. There is no right or wrong.

Please click on "*Next Page*" to start the survey.

**Additional Vaccination**

1. You received the following COVID-19 vaccine. You will find the name of the vaccine on the label in your vaccination certificate:

**PROG**: Allow only one response option. Only appears if "COVID-19" was selected in the registration in question 10.

| BNT162b2 (BioNTech/Pfizer) | ⭘ |
| --- | --- |
| mRNA-1273 (Moderna) | ⭘ |
| ChadOx1 (AstraZeneca) | ⭘ |
| Ad26.COV2.S (Johnson & Johnson) | ⭘ |
| None, appointment cancelled | ⭘ |

1. What was the reason for cancelling the appointment? Multiple-choice answers are possible.

**PROG**: Multiple-choice answers are allowed. Appears only if "None, appointment cancelled" was selected in question 1.

| COVID-19 infection after vaccination | ⭘ |
| --- | --- |
| Intolerance to vaccination | ⭘ |
| Other | ⭘ |

**PROG**: Free text field. Only appears if “Other” is selected in question 2.

1. Did you receive another vaccination since the vaccination at _______?

**PROG**: Allow only single-choice answers. The vaccination selected in the registration in question 10 appears in the placeholder. Only appears if "several" was not selected in the registration in question 10.

| Yes | ⭘ |
| --- | --- |
| No | ⭘ |

1. Did you receive another vaccination since the _______ vaccinations?

**PROG**: Allow only single-choice answers. The vaccinations selected in the registration in question 11 appear in the placeholder. Only appears if "several" was selected in the registration in question 10.

| Yes | ⭘ |
| --- | --- |
| No | ⭘ |

1. You received the following vaccination(s) afterwards. Multiple-choice answers are possible.

**PROG**: Allow multiple response options. Only appears if "Yes" was selected in question 3 or 4.

| Influenza | ⭘ |
| --- | --- |
| Shingles | ⭘ |
| Pneumococcus | ⭘ |
| COVID-19 | ⭘ |
| TBE | ⭘ |
| Tetanus (and/or diphtheria/pertussis/poliomyelitis) | ⭘ |
| None of the above | ⭘ |

1. Have you been diagnosed with COVID-19 disease since your vaccination at _______?

**PROG**: Allow only single-choice answers. The date entered in the registration in question 18 or 21 appears in the placeholder.

| Yes | ⭘ |
| --- | --- |
| No | ⭘ |

**Type of health complaints**

1. Did you experience any health complaints after the ______ vaccination? Please also record complaints that you do not attribute to the vaccination.

**PROG**: Allow only single-choice answers. The placeholder shows the vaccination(s) selected in the registration in question 10 or 11.

| Yes | ⭘ |
| --- | --- |
| No | ⭘ **PROG**: **END** |

1. Which of the following health complaints did you experience? Multiple-choice answers are possible.

**PROG**: Allow multiple-choice answers.

| Complaints at the injection site (e.g. swelling, redness, pain) | ⭘ |
| --- | --- |
| Restricted movement of the arm | ⭘ |
| Pus collection and/or abscess | ⭘ |
| Tiredness and/or fatigue | ⭘ |
| Fever > 38.0°C and/or chills | ⭘ |
| Nausea and/or vomiting | ⭘ |
| Allergic reaction (e.g. skin rash, facial swelling) | ⭘ |
| Shortness of breath | ⭘ |
| Headache | ⭘ |
| Sensory disturbance and/or numbness | ⭘ |
| Circulatory collapse | ⭘ |
| Dizziness | ⭘ |
| Seizure | ⭘ |
| Muscle or joint pain | ⭘ |
| Blood clotting disorder (e.g. thrombosis, embolism) | ⭘ |
| Other | ⭘ |

1. Which other health complaints have you experienced? Please enter your other complaints here:

**PROG**: Free text field. Only appears if "Other" was selected in question 8.

1. Have you ever had an allergic reaction to a vaccine? (e.g. skin rash, shortness of breath, swelling in the throat or face, loss of consciousness).

**PROG**: Allow only single-choice answers. Only appears if "Allergic reaction", "Shortness of breath" or "Circulatory collapse" was selected in question 8.

| Yes | ⭘ |
| --- | --- |
| No | ⭘ |
| I do not know | ⭘ |

1. Has this been your first seizure?

**PROG**: Allow only single-choice answers. Only appears if “Seizure” was selected in question 8.

| Yes | ⭘ |
| --- | --- |
| No | ⭘ |
| I do not know | ⭘ |

**Consequences**

1. Which consequences did your health complaints have? Multiple-choice answers are possible.

**PROG**: Allow multiple-choice answers. Only appears if at least one answer was selected in question 8. A separate question appears for each answer selected in question 8.

| No consequences | ⭘ |
| --- | --- |
| Medication intake | ⭘ |
| Sick leave | ⭘ |
| Consultation (outpatient) | ⭘ |
| Hospital (in-patient/overnight) | ⭘ |
| Hospital (out-patient/hourly) | ⭘ |

Please provide some information about your hospital stay.

1. How many times have you been taken to hospital?

**PROG**: Allow only single-choice answers. Only appears if "Hospital (in-patient/overnight)" and/or “Hospital (out-patient/hourly)” was selected in question 12.

| Once | ⭘ |
| --- | --- |
| Twice | ⭘ |
| 3 times | ⭘ |
| 4 times or more | ⭘ |

1. How long was your (longest) inpatient hospital stay in days?

**PROG**: Allow only single-choice answers. Only appears if "Hospital (inpatient/overnight)" was selected at least once in question12.

| 1 day | ⭘ |
| --- | --- |
| 2 days | ⭘ |
| 3 days | ⭘ |
| 4 days | ⭘ |
| 5 days | ⭘ |
| 6 days | ⭘ |
| 7 days | ⭘ |
| 8 days or more | ⭘ |

1. How did the admission(s) to the hospital take place?

**PROG**: If "Once" was selected in question 13, Allow only single-choice answers. If "Twice" or more was selected in question 13, allow multiple answer choices. Only appears if "Hospital (in-patient/overnight)" and/or “Hospital (out-patient/hourly)” was selected at least once in question 12.

| Referral as a planned intervention by your general practitioner or specialist | ⭘ |
| --- | --- |
| Referral as an emergency by your general practitioner or specialist | ⭘ |
| Referral by a doctor on call | ⭘ |
| Transport by the ambulance service | ⭘ |
| Own presentation in the emergency room | ⭘ |

**Perception**

Please tell us how you felt about your health problems.

**PROG**: Matrix fields.

1. I suspect a connection between my health problems and the vaccination.
2. I feel/felt adversely affected by the health problems.
3. My health problems will have long-term consequences for me.
4. My health problems are comparable to previous vaccinations.

**PROG**: Matrix fields. Allow only single-choice answers per question. Matrix only appears if at least one complaint has been selected in question 8.

| Strongly disagree | ⭘ |
| --- | --- |
| Disagree | ⭘ |
| Agree | ⭘ |
| Strongly agree | ⭘ |

1. With which health complaints do you suspect a connection with the vaccination? Multiple-choice answers are possible.

**PROG**: Allow multiple-choice answers. Each complaint selected in question 8 appears and can be checked on if a connection with vaccination is suspected.

**End of short-term survey 1b**

You have now reached the end of the survey.

Thank you very much for your responses.

Please click on “Submit” to end the survey.

**Long-term survey**

Thank you for taking part.

Mark the answer that most closely matches your assessment. There is no right or wrong.

Please click on "Next Page" to start the survey.

**Medical care**

1. Did you receive another vaccination since the last survey?

**PROG**: Allow only single-choice answers.

| Yes | ⭘ |
| --- | --- |
| No | ⭘ |

1. You received the following vaccination(s) afterwards. Multiple-choice answers are possible.

**PROG**: Allow multiple response options. Only appears if "Yes" was selected in question 1.

| Influenza | ⭘ |
| --- | --- |
| Shingles | ⭘ |
| Pneumococcus | ⭘ |
| COVID-19 | ⭘ |
| TBE | ⭘ |
| Tetanus (and/or diphtheria/pertussis/poliomyelitis) | ⭘ |
| None of the above | ⭘ |

1. Have you been diagnosed with COVID-19 disease since the last survey?

**PROG**: Allow only single-choice answers.

| Yes | ⭘ |
| --- | --- |
| No | ⭘ |

While answering please refer to the period since the last interview (or at least 2 weeks after the last vaccination).

1. Have you seen a doctor because of complaints? Please also refer to complaints that you do not attribute to the vaccination.

**PROG**: Allow only single-choice answers.

| Yes | ⭘ |
| --- | --- |
| Not yet, appointment planned | ⭘ |
| No | ⭘ **PROG**: **END** |

1. Have you been hospitalised due to any complaints? Please also refer to complaints that you do not attribute to the vaccination.

**PROG**: Allow only single-choice answers.

| Yes | ⭘ |
| --- | --- |
| Not yet, admission planned | ⭘ |
| No | ⭘ **PROG**: **END** |

1. Have you had one or more complaints that led to a doctor's examination and/or hospitalisation? Page 1 of 4. Multiple-choice answers are possible.

**PROG**: Allow more than one answer. Only appears if "Yes" or "Not yet, appointment/admission planned" was selected in question 4. and/ or 5.

| Headache | ⭘ |
| --- | --- |
| Dizziness | ⭘ |
| Sensory disturbance | ⭘ |
| Unconsciousness | ⭘ |
| Neuralgia | ⭘ |
| Seizure | ⭘ |
| Epilepsy | ⭘ |
| Stroke/ischemic infarction (apoplexy) | ⭘ |
| Minor stroke (TIA) | ⭘ |
| Paralysis of the facial nerve (facial paresis) | ⭘ |
| Multiple sclerosis | ⭘ |
| None of the above | ⭘ |

1. Have you had one or more complaints that led to a doctor's examination and/or hospitalisation? Page 2 of 4. Multiple-choice answers are possible.

**PROG**: Allow more than one answer. Only appears if "Yes" or "Not yet, appointment/admission planned" was selected in question 4. and/ or 5.

| Diabetes | ⭘ |
| --- | --- |
| Tachycardia/arrhythmia | ⭘ |
| Chest pain | ⭘ |
| Heart attack | ⭘ |
| Peri/myocardial infarction | ⭘ |
| Vascular inflammation (vasculitis) | ⭘ |
| Pulmonary embolism | ⭘ |
| Blood clot (thrombosis) | ⭘ |
| Coagulation disorder | ⭘ |
| None of the above | ⭘ |

1. Have you had one or more complaints that led to a doctor's examination and/or hospitalisation? Page 3 of 4. Multiple-choice answers are possible.

**PROG**: Allow more than one answer. Only appears if "Yes" or "Not yet, appointment/admission planned" was selected in question 4. and/ or 5.

| Muscle weakness | ⭘ |
| --- | --- |
| Back pain | ⭘ |
| arm, leg pain | ⭘ |
| Joint swelling | ⭘ |
| Joint inflammation (arthritis) | ⭘ |
| Muscle twitching | ⭘ |
| Movement disorder | ⭘ |
| None of the above | ⭘ |

1. Have you had one or more complaints that led to a doctor's examination and/or hospitalisation? Page 4 of 4. Multiple-choice answers are possible.

**PROG**: Allow more than one answer. Only appears if "Yes" or "Not yet, appointment/admission planned" was selected in question 4. and/ or 5.

| Flu-like symptoms | ⭘ |
| --- | --- |
| Shortness of breath | ⭘ |
| Fever | ⭘ |
| Nausea/vomiting | ⭘ |
| Abdominal pain | ⭘ |
| Fatigue | ⭘ |
| Feeling of faintness | ⭘ |
| Feeling ill | ⭘ |
| None of the above | ⭘ |

1. If other complaints have occurred, please describe them here:

**PROG**: Free text field. Only appears if "Yes" or "Not yet, appointment/admission planned" was selected in question 4. and/or 5.

1. You are presented with an overview of the complaints you have indicated. Which symptoms did you already suffer from before the vaccination? Multiple-choice answers are possible.

**PROG**: Allow multiple-choice answers. All the complaints selected in question 6, 7, 8 or 9 appear and can be checked in each case if they were already present before the vaccination.

**Perception**

Please tell us how you felt about your health complaints.

**PROG**: Matrix fields.

1. I suspect a connection between my health complaints and the vaccination.
2. I feel/felt adversely affected by the health complaints.
3. My health complaints will have long-term consequences for me.
4. My health complaints are comparable to previous vaccinations.

**PROG**: Matrix fields. Allow only single-choice answers. Matrix only appears if at least one complaint has been selected in question 8.

| Strongly disagree | ⭘ |
| --- | --- |
| Disagree | ⭘ |
| Agree | ⭘ |
| Strongly agree | ⭘ |

1. With which health complaints do you suspect a connection with the vaccination? Multiple-choice answers are possible.

**PROG**: Allow multiple-choice answers. Each complaint selected in question 6 - 10 appears and can be checked on if a connection with vaccination is suspected.

**Medical report**

Main diagnosis in the medical report.

1. You have almost finished. If you have a doctor's letter for the health complaints mentioned, you canenter your main diagnosis(es).

**PROG**: Free text field. Only appears if at least one complaint was selected in question 6, 7, 8 or 9.

**End of long-term survey**

You have now reached the end of the survey.

Thank you very much for your responses.

Please click on “Submit” to end the survey.
